# Multi-parent advanced generation inter-cross (MAGIC) populations in rice: progress and potential for genetics research and breeding

**DOI:** 10.1186/1939-8433-6-11

**Published:** 2013-05-06

**Authors:** Nonoy Bandillo, Chitra Raghavan, Pauline Andrea Muyco, Ma Anna Lynn Sevilla, Irish T Lobina, Christine Jade Dilla-Ermita, Chih-Wei Tung, Susan McCouch, Michael Thomson, Ramil Mauleon, Rakesh Kumar Singh, Glenn Gregorio, Edilberto Redoña, Hei Leung

**Affiliations:** Division of Plant Breeding, Genetics and Biotechnology, International Rice Research Institute, Manila, Philippines; Department of Plant Breeding and Genetics, Cornell University, Ithaca, NY USA; Department of Agronomy, National Taiwan University, Taipei, Taiwan

**Keywords:** Multi-parent Advanced Generation Inter-Crosses (MAGIC), Genotyping-By-Sequencing (GBS), Genome-Wide Association Study (GWAS)

## Abstract

**Background:**

This article describes the development of Multi-parent Advanced Generation Inter-Cross populations (MAGIC) in rice and discusses potential applications for mapping quantitative trait loci (QTLs) and for rice varietal development. We have developed 4 multi-parent populations: *indica* MAGIC (8 *indica* parents); MAGIC plus (8 *indica* parents with two additional rounds of 8-way F1 inter-crossing); *japonica* MAGIC (8 *japonica* parents); and Global MAGIC (16 parents – 8 *indica* and 8 *japonica*). The parents used in creating these populations are improved varieties with desirable traits for biotic and abiotic stress tolerance, yield, and grain quality. The purpose is to fine map QTLs for multiple traits and to directly and indirectly use the highly recombined lines in breeding programs. These MAGIC populations provide a useful germplasm resource with diverse allelic combinations to be exploited by the rice community.

**Results:**

The *indica* MAGIC population is the most advanced of the MAGIC populations developed thus far and comprises 1328 lines produced by single seed descent (SSD). At the S4 stage of SSD a subset (200 lines) of this population was genotyped using a genotyping-by-sequencing (GBS) approach and was phenotyped for multiple traits, including: blast and bacterial blight resistance, salinity and submergence tolerance, and grain quality. Genome-wide association mapping identified several known major genes and QTLs including *Sub1* associated with submergence tolerance and *Xa4* and *xa5* associated with resistance to bacterial blight. Moreover, the genome-wide association study (GWAS) results also identified potentially novel loci associated with essential traits for rice improvement.

**Conclusion:**

The MAGIC populations serve a dual purpose: permanent mapping populations for precise QTL mapping and for direct and indirect use in variety development. Unlike a set of naturally diverse germplasm, this population is tailor-made for breeders with a combination of useful traits derived from multiple elite breeding lines. The MAGIC populations also present opportunities for studying the interactions of genome introgressions and chromosomal recombination.

**Electronic supplementary material:**

The online version of this article (doi:10.1186/1939-8433-6-11) contains supplementary material, which is available to authorized users.

## Background

Breeders and molecular geneticists have routinely used populations derived from bi-parental crosses for variety development and mapping quantitative trait loci (QTLs) for traits of interest. Most of the varieties developed by breeders in self-pollinated crops like rice are based on single crosses between two parents. Breeders have also attempted to make multiple crosses (e.g., three-way involving three parents, or double crosses involving four parents) to increase the genetic variation in breeding populations; however, extensive use of these multiple crosses may be restricted by technical limitations (e.g., intensive labour for crossing and large population sizes required for recovering recombinants with all the desirable traits). More complex crossing schemes involving 6-way, 8-way crosses or diallel selective mating for use in breeding of self - pollinated crops were proposed many years ago (Allard 1960; Jensen 1970) but have seldom been used in plant breeding programs.

Conventional QTL mapping is used to identify the location and effects of QTLs controlling traits of interest. Typical populations used for QTL mapping include F_2_, backcross (BC) or recombinant inbred (RI) populations derived from only two parents. The limitations of using such bi-parental populations are that only two alleles are analyzed and that genetic recombination in these populations is limited (especially in F_2_ or BC populations) which limits the resolution for QTL detection. Most bi-parental populations have only one opportunity for crossing over and about 34 break-up points are estimated to occur per crossing generation (Huang et al., 2009). Recently, a multi-parent advanced generation intercross (MAGIC) strategy has been proposed to interrogate multiple alleles and to provide increased recombination and mapping resolution (Cavanagh et al. 2008). The main objective of developing MAGIC populations is to promote intercrossing and shuffling of the genome. The advantages of using multi-parent populations are that: (1) more targeted traits from each of the parents can be analyzed based on the selection of parents used to make the multi-parent crosses; and (2) increased precision and resolution with which QTLs can be detected due to the increased level of recombination (Cavanagh et al. 2008). Multi-parent populations are now attractive for researchers due to the development of high-throughput SNP genotyping platforms and advances in statistical methods to analyze data from such populations.

Advanced Inter-crossed Lines (AILs) are the fixed populations serving as permanent resources derived from MAGIC and are similar to recombinant inbred (RI) populations derived from bi-parental crosses. AILs are generated by randomly and sequentially inter-crossing a population initially originating from a cross between two inbred lines (Darvasi and Soller 1995); however, in our case multiple parents are used. Advanced intercrosses (AIC), which are derived from multiple parents, have increased recombination events in small chromosomal regions and can be used for fine mapping (Huang and George 2011). Each generation of random mating reduces the extent of linkage disequilibrium (LD), allowing the QTL to be mapped more precisely (Rockman and Kruglyak 2008). Lines derived from early generations can be used for QTL detection and coarse mapping, while those derived from later generations may potentially yield markers very close to the QTL due to increased crossing-over events after every inter-mating cycle. This allows parallel high-resolution mapping of different complex traits in the same population. For example, the complex trait consortium developed eight-way crosses in mice to explore the genomes of the recombinant inbreds, as discussed in detail by Broman (2005). Association analysis in the collaborative cross (CC) in mouse shows that they have achieved highly diverse CC inbreds with allelic balance from the founders and that recombination sites were distributed uniformly, which is ideal for genetic studies (Aylor et al. 2011).

In plants, MAGIC populations were first developed and described in *Arabidopsis* (Cavanagh et al., 2008; Huang et al., 2011; Kover et al., 2009). Cavanagh et al. (2008) discuss in detail the production of the MAGIC population, methods of mapping genes to traits and the relevance of such population to breeders. In *Arabidopsis* a MAGIC population was derived from inter-crossing of 19 accessions and mapping of quantitative traits detected QTLs contributing to low levels of phenotypic variation (Kover et al. 2009). *Arabidopsis* multi-parent RIL (AMPRIL) populations were genotyped at the F_4_ stage and phenotyped at F_5_ and mixed-models were used for fine mapping (Huang et al. 2011). MAGIC populations are also being actively developed in various plant species (https://sites.google.com/site/ijmackay/work/magic). More recently, Huang et al. (2012) demonstrated the use of a large MAGIC wheat population for mapping QTLs underlying complex traits such as plant height and hectolitre weight. The increased recombination in MAGIC populations can lead to novel rearrangements of alleles and greater genotypic diversity.

**Figure 1:**
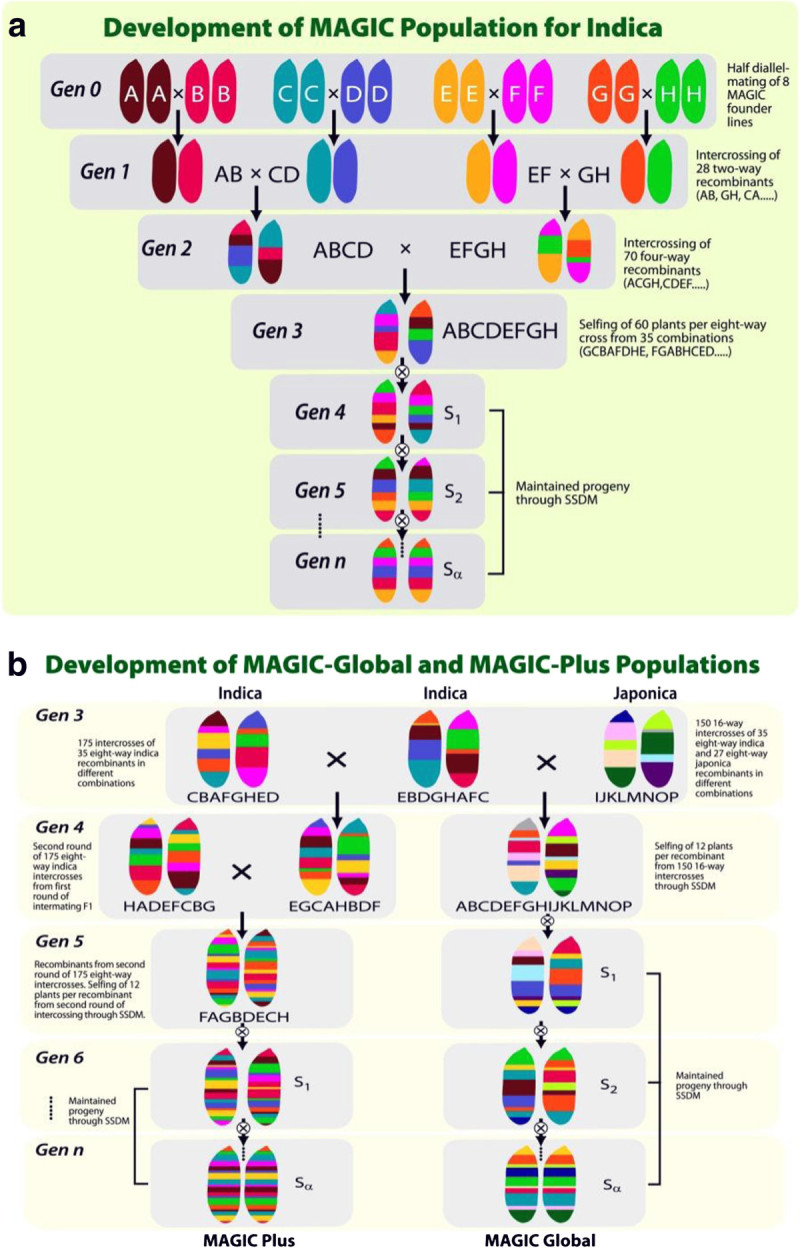
**Crossing schemes to produce four multi-parent advanced generation inter-cross (MAGIC) populations: a) Development of the** ***indica*** **MAGIC population.** The same scheme was used for the development of the *japonica* MAGIC population **b**) Development of the MAGIC plus and global MAGIC populations. The global population includes eight *indica* and eight *japonica* founder lines, which carry QTLs conferring tolerance of biotic and abiotic stresses. A, B, C, D, E, F, G and H – represent the 8 *indica* parents; I, J, K, L, M, N, O and P – represent the 8 *japonica* parents; Gen- generation; SSDM – single seed descent method ,S – selfing.

Given the potential benefits of MAGIC populations, we initiated the development of MAGIC populations using the two major rice ecotypes: *indica* and *japonica*. We aimed to produce at least 2,000 inbred lines to capture the broadest genotypic diversity through inter-crossing of 8 *indica* parents for *indica* MAGIC and 8 *japonica* parents for the *japonica* MAGIC population. As the project progressed, we produced an *indica* population with two more cycles of inter-crossing to enhance recombination, referred to as the MAGIC plus population. Finally, we inter-crossed the *indica* and *japonica* base populations to increase the overall diversity. This population was called the “global MAGIC” population as it encompasses the *indica* and *japonica* rice ecotypes that are grown globally (Figure 1a and b). We chose 8 *indica* and 8 *japonica* founder lines based on breeding relevance to develop the MAGIC populations (Table 1). We only included improved and adapted breeding lines or varieties as donors for one or more traits rather than land races. This was to eliminate the chance of carrying over undesirable traits in recombinants. These founder lines possess desirable agronomic traits as well as disease resistance and tolerance to abiotic stresses. Some of the traits that are considered essential across environments include yield, drought tolerance, tolerance to salinity, submergence tolerance, and resistance to blast and bacterial blight diseases.

**Table 1:** **Agronomic relevance of the 16 founder lines used in developing the** ***indica*** **and** ***japonica*** **MAGIC populations**

Germplasm/variety	GID^1^	Varietal type	Origin	Agronomic relevance
**Indica type**				
Fedearroz 50	1846419	*Indica*	Colombia	Popular variety in several countries, with stay green/delayed senescence & quality traits, disease tolerance, progenitor of many breeding lines
Shan-Huang Zhan-2 (SHZ-2)	402862	*Indica*	China	Blast resistant, high yielding; in the pedigrees of many varieties in south China
IR64633-87-2-2-3-3 (PSBRc82)	94801	*Indica*	IRRI	High yielding and most popular variety of the Philippines
IR77186-122-2-2-3 (PSBRc 158)	1111266	*Indica* / tropical *japonica* background	IRRI	High yielding variety in New Plant Type II background
IR77298-14-1-2-10	2154106	*Indica*	IRRI	Drought tolerant in lowlands with IR64 background and tungro resistance
IR4630-22-2-5-1-3	56023	*Indica*	IRRI	Good plant type, salt tolerant at seedling and reproductive stages
IR45427-2B-2-2B-1-1	1935108	*Indica*	IRRI	Fe toxicity tolerant
Sambha Mahsuri + Sub1	2254836	*Indica*	IRRI	Mega variety with wide compatibility, good grain quality and submergence tolerance
**Japonica group**				
CSR 30	1158955	Basmati group	India	Sodicity tolerance; Basmati type long aromatic grain
Cypress	417083	Tropical japonica	USA	High yielding, good grain quality and cold tolerant
IAC 165	599974	Tropical japonica	Latin America	Aerobic rice adaptation
Jinbubyeo	312160	Temperate japonica	Korea	High yielding and cold tolerant
WAB 56-125	94428	O. glaberrima in *indica* background	WARDA	NERICA background *(O. glaberrima*); heat tolerant and early flowering
IR73571-3B-11-3-K2	2007669	Cross between tropical japonica and indica	IRRI-Korea project	Tongil type, salinity tolerant
Inia Tacuari	1846418	Tropical japonica	Uruguay	With earliness, wide adaptation, & good grain quality
Colombia XXI	2351848	Tropical japonica	Colombia	High yielding and delayed senescence

^1^*GID*, Germplasm identification.

Here, we report on the status of the development of MAGIC populations in rice and results from genotypic and phenotypic analyses of a subset of the *indica* MAGIC population. Analysis of the S4 generation of the *indica* MAGIC population detected major QTLs for biotic and abiotic traits transmitted from the parents, and demonstrated the potential power of this approach for genetic studies and breeding applications.

## Results and discussion

### Developing multiple MAGIC populations in rice

Two initial MAGIC populations have been developed by inter-crossing eight elite lines from the Asia *indica* pool (*indica* MAGIC) and the *japonica* group (*japonica* MAGIC). Each population is comprised of 8 founder lines that include elite and modern varieties known to exhibit high yield potential, good grain quality, and tolerance to a range of biotic and abiotic stresses (Table 1).

The *indica* and *japonica* MAGIC populations followed the same scheme of development. The first stage followed a half-diallele mating system by inter-mating the eight founder lines and 28 bi-parental crosses were made. The resulting 28 F_1_’s were inter-crossed to derive 4-way crosses for which 70 such 4-way crosses (out of 210 possible crosses) were made. The 70 crosses were selected in such a manner that no parent was represented more than once in the 4-way cross. Also only one of the possible 4-way combinations was selected (e.g., one of ABCD or ACBD or BCAD etc.). The last stage involved intercrossing of the 70 4-way crosses to derive 8-way crosses. Only 35 out of 105 such possible 8-way crosses were made keeping in mind that no parent was represented more than once. From each of the 35 8-way crosses ~60 seeds were advanced by selfing. Single plant selections were made to advance to the next generation. Thereby the population size targeted was 35 × 60 = 2100 lines. A pedigree for a line in the MAGIC *indica* population would for example be written as A/C//E/H///B/F//D/G.

In the case of the MAGIC plus population the 8-way crosses derived during the development of the *indica* MAGIC population underwent two extra-rounds of inter-crossing prior to selfing. A set of 175 multiple crosses were made for each of the inter-crossing rounds. The pedigree for a line derived from the MAGIC plus population would for example be written as (H/E//A/F///C/D//B/E/4/A/E//F/C///B/H//D/G/5/C/H//E/F///A/B//D/G/4/H/E//G/A///D/C//B/F).

The Global MAGIC population was developed by crossing the 8-way crosses derived during the development of the *indica* MAGIC population to the 8-way crosses derived during the development of the *japonica* MAGIC population. A total of 150 such multiple crosses were made. Therefore the Global MAGIC population is representative of 16 parents (8 *indica* type and 8 *japonica* type). The 16-way crosses were then advanced by selfing.

The *indica* MAGIC population consists of 2000 lines advanced by single seed descent (SSD) of which currently 1328 are in the S7 generation and the remaining lines are following as they are late maturing. The *japonica* MAGIC population is smaller relative to the *indica* MAGIC population, consisting of 500 lines at the S5 stage. This population will be suitable to temperate environments, and these lines will initially be tested for their performance in Korea. The MAGIC plus population is currently at the S4 stage and is potentially more valuable due to the added round of inter-crossing. This population is expected to further extend the amount of recombination when compared to the *indica* MAGIC population. We hope to address whether the extra two rounds of inter-crossing increased the levels of recombination enough to enable direct fine-mapping of QTLs using the MAGIC population. The Global MAGIC is an attempt to combine traits from both *indica* and *japonica* gene pools that have been adapted to different environments. In addition to being a source of potential novel variation, the Global MAGIC population provides useful materials for studying the relative contributions from the two rice ecotypes and the level of recombination among 16 genomes constituting this population. The crossing schemes for the different MAGIC populations are shown in Figure 1a and 1b.

### Analysis of a subset of *indica* MAGIC population

**Figure 2:**
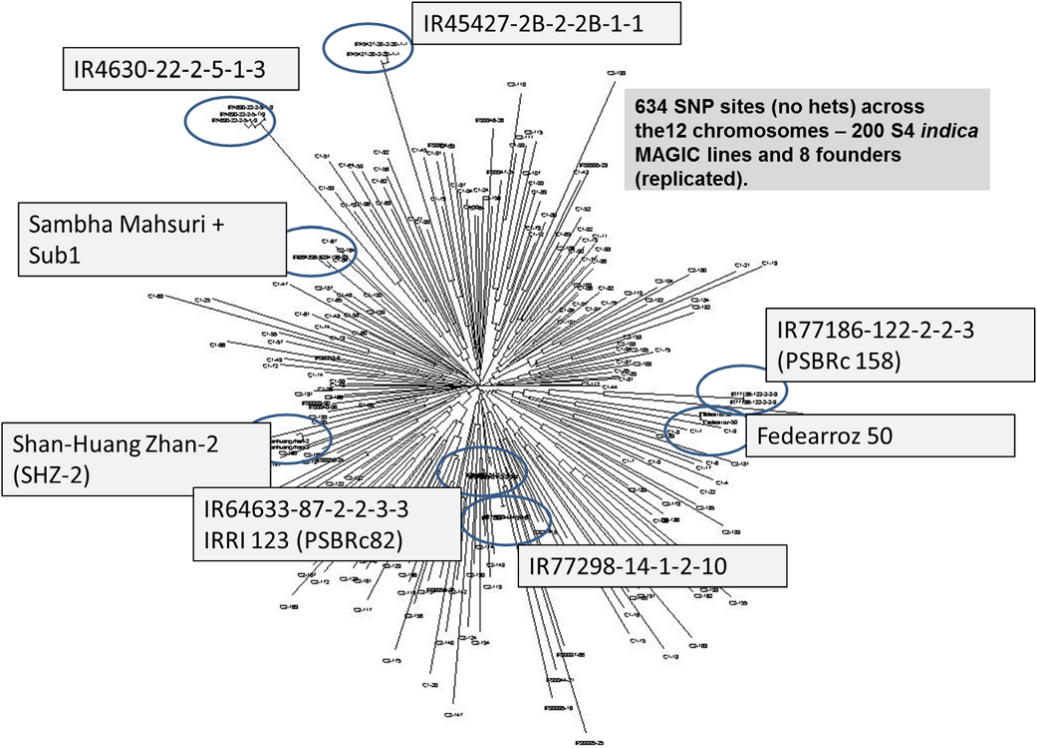
**Cladogram (neighbor joining) of the 200 S4** ***indica*** **MAGIC lines and 8 founders using 634 SNP marker sites (no missing calls or heterozygous sites).**

In order to evaluate the utility of MAGIC population for QTL mapping and breeding, a subset of 200 S4 SSD lines along with the 8 founders of the *indica* MAGIC population was phenotyped for resistance to biotic stresses (blast disease, bacterial blight - strains PXO61, PXO86, PXO99 and PXO341) and abiotic stresses (salinity, submergence) and grain quality. For genotyping, a high resolution approach was needed to take advantage of the highly recombined population. Single nucleotide polymorphism (SNP) markers are the most abundant types of DNA markers and are the marker of choice due to the availability of extensive rice genome sequences and the development of low cost SNP genotyping approaches. Recently, genotyping by sequencing (GBS) has been used as an efficient method to provide high resolution SNP genotyping through restriction digestion and multi-plexed sequencing (Elshire et al. 2011). To test the efficacy of using GBS for high-resolution mapping of the MAGIC populations, the *indica* subset and the founders were genotyped using a 96-plex *Ape* KI GBS protocol (see Methods). This GBS technology provided 17,387 polymorphic SNP marker sites across all 12 chromosomes with a call rate >60% and a minor allele frequency > 0.05. We used the Trait Analysis by aSSociation, Evolution and Linkage (TASSEL) program (Bradbury et al. (2007, 2011) to perform a Genome-Wide Association Study (GWAS) using the phenotypic and genotypic data on the subset of 200 *indica* MAGIC lines. The mixed linear model (MLM), which accounts for population structure was used for GWA mapping. Although population structure analysis in TASSEL shows that the *indica* MAGIC population used for GWAS has negligible structure (Figure 2) results from both models were compared. In order to detect the levels of false positives in the GLM output we used q-values which measure the false discovery rate of calling the marker significant. Cladogram of the 200 lines shows negligible structure (Figure 2) but in order to account for it the MLM analysis was conducted. SNPs showing significant associations by MLM are discussed in the results. Manhattan plots from MLM analysis have been used to show significant associations that were detected in this study (Manhattan plots from GLM analysis, Additional file 1) and a list of selected significant SNP markers from MLM analysis (Additional file 2) for each of the traits is also provided.

### Biotic stress

#### Blast disease

Blast disease, caused by *Magnaporthe oryzae*, is one of the most important fungal diseases of rice, causing significant yield losses in many rice producing areas. Shan-Huang Zhan-2 (SHZ-2) was selected as the donor parent as it has been widely used in breeding for resistance to blast disease. SHZ-2 exhibits broad spectrum resistance to blast disease and mapping studies have detected three contributing QTLs on chromosomes 2, 6 and 9 (Liu et al. 2011). To date, a number of *Pi* genes contributing to the resistance of blast disease have been identified and cloned with the most important ones being located on chromosomes 1, 2, 6, 8, 11 and 12 (Ballini et al. 2008). The subset of 200 *indica* MAGIC lines and founders were screened for blast disease against natural isolates in the blast nursery at IRRI, Philippines. Out of the 8 founders, 6 (PSBRc 82, Fedearroz 50, IR 4630-22-2-5-1-3, Sanhuangzhan-2, PSBRc 158 and Samba Mahsuri-Sub1) were resistant to blast disease under natural conditions in the blast nursery at IRRI. Screening results suggest the transmission of blast resistance from the donors to a majority of the S4 progeny.

**Figure 3:**
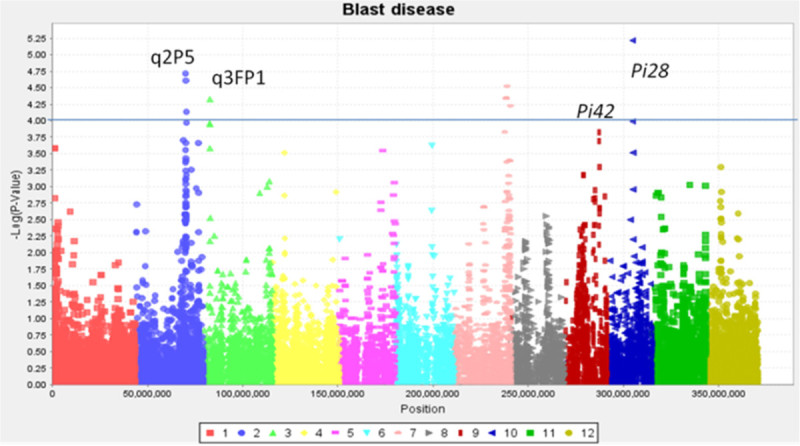
**Blast disease** - **Manhattan plot (MLM) showing GWA and highlighting significant associations near previously detected QTLs on chromosomes 2, 3, 9 and 10; x axis** – **position on chromosomes 1 to 12; y**-**axis** **(-) Log p**-**value of markers; solid line** – **p < 0.0001.**

MLM analysis identified 10 significant markers (p < 0.0001) on chromosomes 2, 3, 7 and 10. The association on chromosome 3 was around 3.5 Mb near q3FP1 (based on field screening) reported by Ballini et al. (2008). Associations were detected on chromosome 7 (27 Mb) and chromosome 2 around 26 Mb which aligns with meta QTL q2P5 (Ballini et al. 2008). This peak was also detected within a cluster of 23 SNP markers by GLM analysis (p < 0.0001 and q < 0.05). GLM (p < 0.0001 and q <0.05) and MLM (p < 0.0002) analysis detected significantly associated markers on chromosome 9 at 18 Mb near *Pi42* and on chromosome 10 at 13Mb near *Pi28* (Ballini et al. 2008) (Figure 3).

#### Bacterial blight

Bacterial blight caused by *Xanthomonas oryzae* is the most important and widespread bacterial disease of rice in irrigated and rainfed ecosystems. There are several races which occur in specific geographic regions. PSBRc82, a very popular variety in the Philippines, carries the *Xa4* dominant gene on chromosome 11 conferring resistance to Philippine races 1, 4, 5, 7, 8 and 10 (Nino-Liu and Ronald, 2006). The subset of *indica* MAGIC lines and the 8 founders were screened against Race 1 (PXO61), Race 2 (PXO86), Race 6 (PXO99) and Race 10 (PXO341) at maximum tillering stage in the screen house at IRRI. Results indicate that majority of the 200 lines carried the *Xa4* gene.

**Figure 4:**
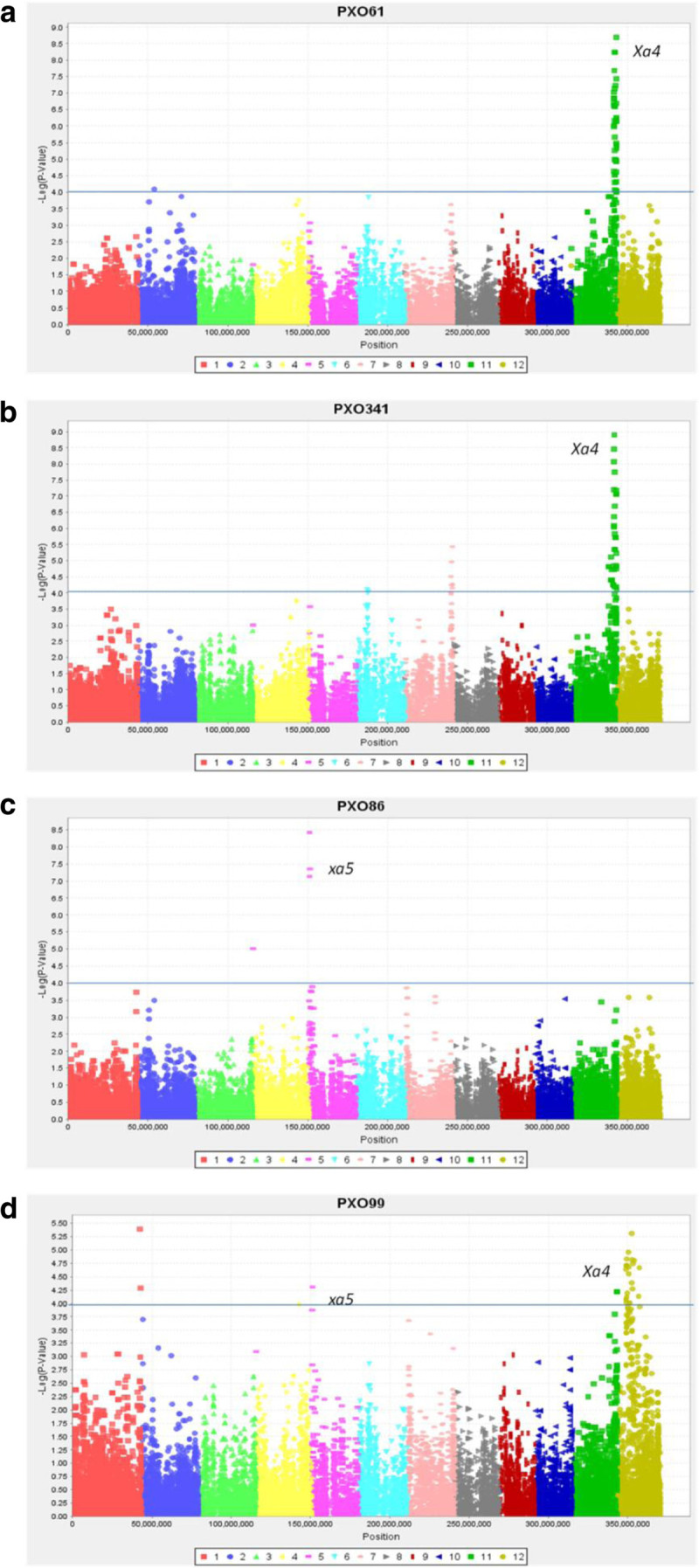
**Bacterial blight - Manhattan plots (MLM) showing GWA to bacterial blight strains (a) PXO61, (b) PXO341, (c) PXO86, and (d) PXO99.** Note the high level of association with SNPs on chromosome 11 (figures a and b) pointing to *Xa4* and *xa5* on chromosome 5 (figure c). GWAS detects both *Xa4* and *xa5* (figure d) in response to PXO99. x axis – position on chromosomes 1 to 12; y-axis (-) Log p-value of markers; solid line – p < 0.0001.

For Race 1 (strain PXO61), MLM analysis (p < 0.0001) detected 52 significantly associated SNP markers, with a larger cluster of 51 markers on chromosome 11 (physical position 27.7 Mb; marker R^2^ = 0.28) at the position of the *Xa4* gene (Wang et al. 2001) (Figure 4a).

In the case of Race 10 (strain PXO341), MLM analysis detected 49 significant SNP markers with the major cluster (40 markers) flanking *Xa4* on chromosome 11 (physical position 27.3 Mb - 27.9 Mb) (Figure 4b). Both MLM (p < 0.0001) and GLM (p < 0.0001 and q < 0.05) also detected a significant association to PXO341 on chromosome 7 around 28–29 Mb.

Thus, GWAS results using GBS indicated a resistance gene against races PXO61 and PXO341 on chromosome 11 (Figure 4b) at 27.2 - 28.4 Mb, which is flanking *Xa4* at 27.7 Mb, providing confirmation that the GBS data can clearly identify the location of significant QTLs, even with just 200 MAGIC lines.

In addition, GWAS results from this study also detected the *xa5* gene on chromosome 5 (physical position 0.4 Mb) in the population. The *xa5* gene is a recessive gene and confers resistance to Philippines races 1, 2, 3, 5, 7, 8, 9 and 10 (Nino-Liu and Ronald 2006).

MLM analysis (p < 0.0001) to Race 2 (strain PXO86) detected 10 associated SNP markers. The markers detected on chromosome 5 (physical position 0.44 Mb; marker R^2^ =0.17, Figure 4c) were identified exactly at the position of the *xa5* gene (Iyer and McCouch 2004). Although *xa5* is effective against races PXO61 and PXO341 it is possible that *Xa4* masked the effect of *xa5* which explained why we were unable to detect an association with *xa5* using these two strains. This explanation is reasonable because when the population was tested with PXO86 against which *Xa4* is not effective, we detected the effect of *xa5.* Further experiments involving mapping populations segregating for *Xa4* and *xa5* would be needed to test this hypothesis.

MLM analysis (p < 0.0001) to Race 6 (strain PXO99) detected 29 significant makers largely on chromosome 12 (5–14 Mb). Although *Xa4* and *xa5* are not effective by themselves (or in combination) against this race (Cottyn and Mew 2004), it is possible that there were interactions with other QTLs on chromosomes 1 or 12, which were more significant relative to *Xa4* or *xa5*. This may account for the detection of significant associations on chromosome 11 at 28.2 Mb near *Xa4* and the associated markers on chromosome 5 near *xa5* (0.44 Mb) (Figure 4d). The identification of two putatively novel QTLs and their possible interactions with other *Xa* genes needs further validation in mapping populations segregating for *Xa4*, *xa5*, QTL-1 and QTL-12.

### Abiotic stress

#### Salt tolerance

**Figure 5:**
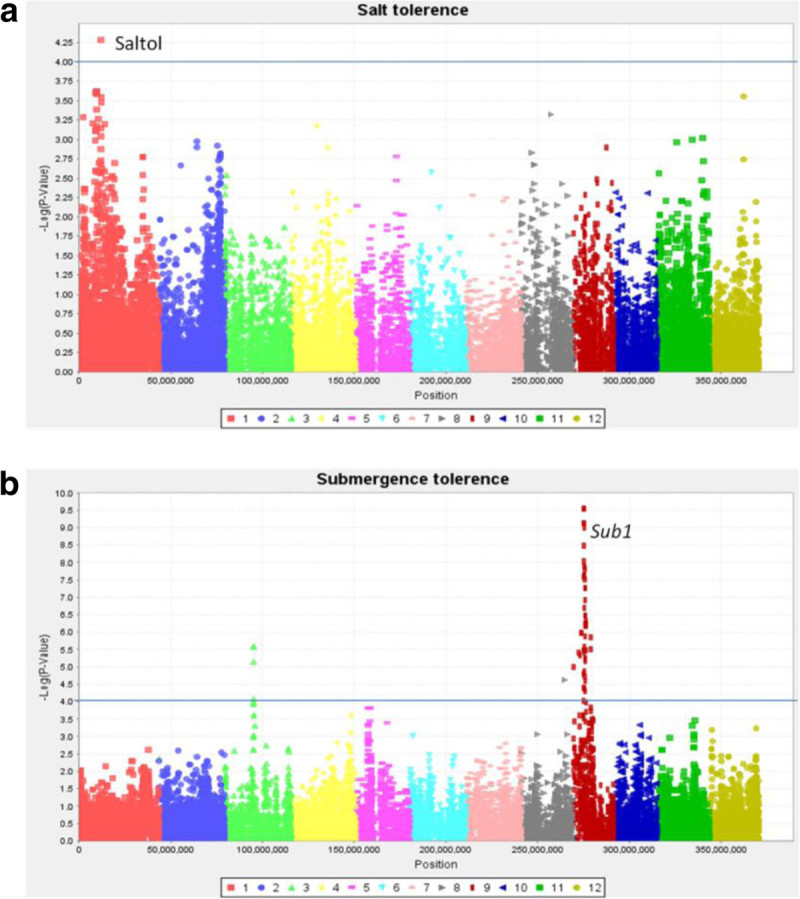
**Abiotic stress** - **Manhattan plots** **(MLM)** **showing GWA to** **(a)** **salt tolerance and** **(b)** **submergence.** Note the association on chromosome 1 - Saltol to salt tolerance and detection of the *SUB1* locus on chromosome 9 in response to submergence. x axis – position on chromosomes 1 to 12; y-axis (-) Log p-value of markers; solid line – p < 0.0001.

Salinity is a wide-spread abiotic stress especially in the rainfed lowlands near coastal regions. Salinity may constrain rice production due to sensitivity of rice at seedling and reproductive stages (Singh and Flowers, 2010). The subset of the *indica* MAGIC S4 lines and founders were screened for salt tolerance (seedling) in the IRRI phytotron at electrical conductivity (EC) 12 dSm^-1^ (deciSiemens per meter). Results indicated the presence of lines with good levels of tolerance, presumably from the salt tolerant parents IR4630-22-2-5-1-3 or IR45427-2B-2-2B-1-1. MLM analysis (P < 0.0001) detected significant markers on chromosome 1 at 11.8 Mb and at P < 0.0005 significant markers were detected on chromosome 1 between 9.2 and 12 Mb near the previously detected QTLs SALTSN, salt sensitivity, q*SKC-1* and near the Saltol QTL (Alam et al. 2011; Thomson et al. 2010) (Figure 5a).

#### Submergence tolerance

Submergence is another important abiotic stress in rainfed and irrigated ecosystems. Samba Mahsuri-Sub1 was used as the donor parent of the *Sub1* locus. The subset of *indica* MAGIC S4 lines along with parents was screened for submergence tolerance in the submergence plots at IRRI and several lines exhibited a good level of tolerance to submergence comparable to checks. MLM analysis (p < 0.0001) detected 54 associated markers 49 of which were detected on chromosome 9 at 6.2 - 6.3 Mb (Figure 5b) in the region of the *Sub1* locus (Neeraja et al. 2007).

### Grain quality

The eight founder parents represented different quality attributes. Two parents in particular – Samba Mahsuri + Sub1 and PSBRc82 – have highly desirable quality attributes in India/Bangladesh and Philippines, respectively. Grain and cooking quality are essential characteristics of rice varieties and hence several key quality traits were evaluated, including amylose content, grain shape and gelatinization temperature. Amylose content (AC) is a key determinant of cooking quality and consumer preferences. The S4 grains of the same subset of *indica* MAGIC and founders were tested for grain quality at the IRRI Grain Quality Nutrition and Postharvest Center. GWAS detected known major effect QTLs along with several potentially novel QTL for grain quality and grain shape (Bai et al. 2010; Zheng et al. 2008), as described below.

**Figure 6:**
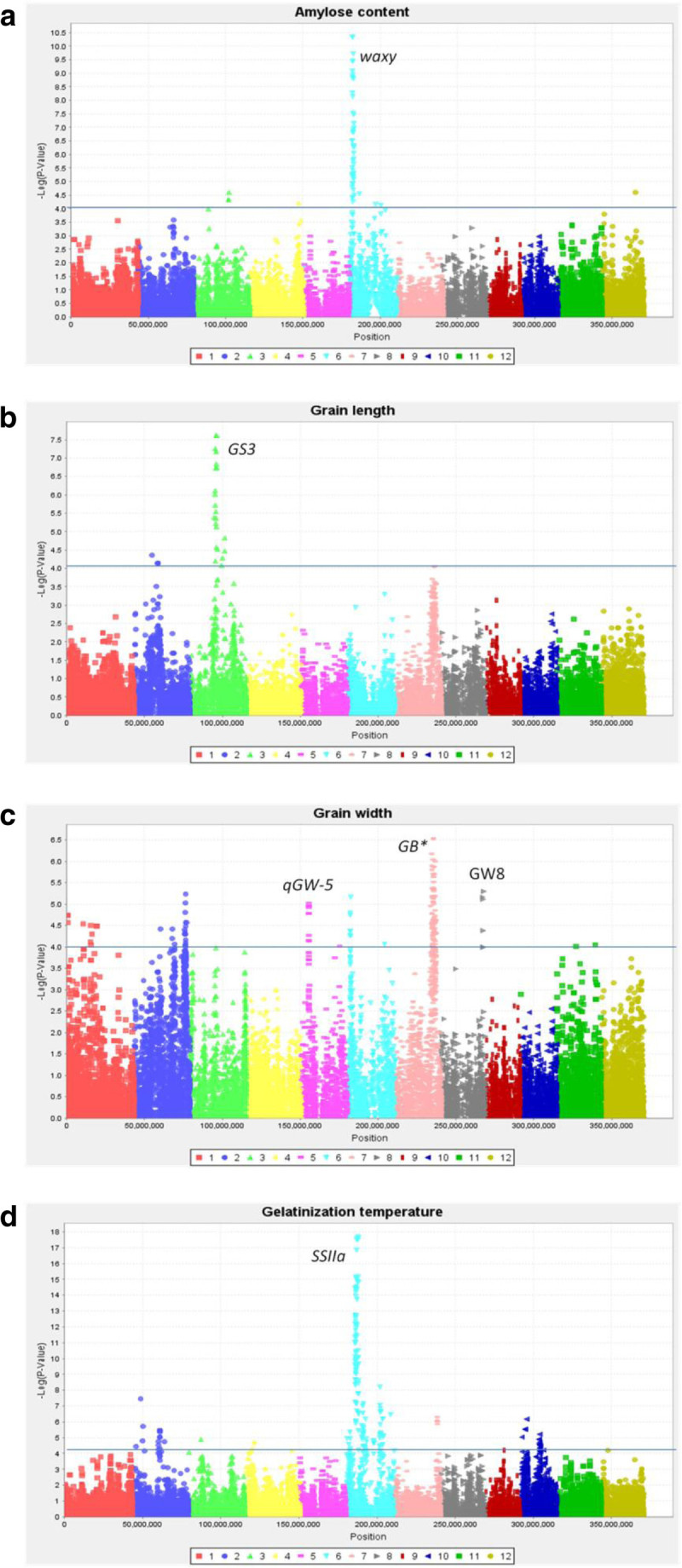
**Grain quality** - **Manhattan plots** **(MLM)** **showing GWA for** **(a)** **amylose content** – ***waxy*** **chromosome 6** **(b)** **grain length GS3 on chromosome 3** **(c)** grain width GB* **QTL for grain breadth Ref.** Redoña and Mackill (1998) on chromosomes 7 and (**d**) gelatinization temperature *SSIIa* Chromosome 6. x axis – position on chromosomes 1 to 12; y-axis (-) Log p-value of markers; solid line – p < 0.0001.

MLM analysis for amylose content detected 61 significant markers with a major cluster on chromosome 6. As expected, the cluster of highly significant SNPs on chromosome 6 was identified at the *waxy* locus on chromosome 6 at 1.7 Mb (Figure 6a) (Zheng et al. 2008).

GWAS analysis for grain length using MLM analysis detected 31 significant SNP marker sites (p < 0.0001) of which 25 were on chromosome 3 (15–22.2 Mb). The highly significant SNP markers detected on chromosome 3 point to the major gene for grain length, *GS3* (Fan et al. 2006) (Figure 6b). In case of grain width MLM analysis (p < 0.0001) detected 130 sites with 73 SNP markers on chromosomes 7 and 4 markers on chromosome 8 (Figure 6c). The significant markers on chromosome 7 (22.7 – 26 Mb) co-localized with the grain breadth QTL identified in the study by Redoña and Mackill (1998) and was near a grain shape QTL *GS7* (Shao et al. 2012). The significant markers on chromosome 8 (26.4 - 27.2 Mb) co-localized with GW8 *OSSPL16* QTL for grain width and yield potential (Wang et al. 2012). MLM also detected associations on chromosome 5 within the fine-mapped region of QTL *qGW-5* (3.5-6.8 Mb) (Weng et al. 2008).

Gelatinization temperature indicates cooking temperature and falls into three categories- high, intermediate, and low. Our data shows that we have all the three classes of gelatinization temperatures ranging from <70 °c to greater than 74 °c. MLM analysis detected 95 SNP makers with a highly significant cluster on chromosome 6 (5.6 – 7.9 Mb) for starch gelatinization temperature which coincided with starch synthase gene (*SSIIa*) (Waters et al. 2006) which is used to assess cooking time or quality (Figure 6d). Significant association on chromosome 2 was also detected. These loci provide exciting new targets for further investigation into the genetic control of grain quality in rice.

### Use of MAGIC lines in breeding programs

As the MAGIC populations were advanced breeders at IRRI made early generation selections from the *indica* MAGIC population and included them in their breeding programs targeting irrigated and rainfed environments. Under these individual breeding programs trait-specific selections have also been made. In order to make the MAGIC population available to the rice breeding community as quickly as possible, an S2:4 bulk population was developed and 400 lines were selected based on agronomic traits. Bulk seeds harvested from the 400 lines have been included in multi-environment trials (MET) with sites in the Philippines and Tanzania. The S2:4 bulk lines were phenotyped for agronomic traits and preliminary results suggest possible transgressive segregation of some complex traits, including yield and drought tolerance, in the population. Based on yield and agronomic performance at three sites in the Philippines (Iloilo, Bukidnon and IRRI) 130 bulked lines have been selected and included in a larger MET in the Philippines, Vietnam, Cambodia, Myanmar, China, Thailand, Tanzania and Senegal. Breeders at these sites have selected lines suited to their environments. Also, breeders of collaborating institutions have visited the field at IRRI and selected MAGIC lines that will be further tested in their countries.

## Conclusions

Each of the eight founder parents were carefully selected as a donor for at least one major trait in an improved background. Interestingly, for many traits, the sources of tolerance or resistance were also identified in other founder parents (i.e. in addition to the selected donor parent). This suggests that that MAGIC populations have potential for identifying useful QTLs from a diverse set of parents. Another major finding of this study was the successful use of GBS in the precise detection of previously-characterized major genes or QTLs for several traits. This implies that the density of SNPs generated by using GBS was sufficient for reliable association mapping in a relatively small mapping population. Many potentially novel QTLs were also identified which could be of great value for further elucidating the molecular genetic control of the traits investigated, as well as to provide recombined lines with multiple traits for breeding programs. These minor QTLs will be validated in independent populations and environments to confirm their effects. Those with significant associations can be further validated in larger populations to better define their positions and enable comparisons with underlying candidate genes. Our results also highlight the potential use of GBS for QTL mapping using multi-parent populations in other plant species in the future. A list of markers significantly associated with individual trait is shown in Additional file 2.

The MAGIC populations can also be explored to study gene-gene interactions. This maybe more challenging as the background is a complex mix of 8 parents. For instance a QTL may not be effective in a single background (one of the eight parents), but maybe be effective in the MAGIC populations (a mixed background of the 8 parents).

The preliminary examination of the S4 SSD *indica* MAGIC population suggests that it is possible to capture various combinations of traits in the population (Table 2), which is directly applicable in breeding programs. We plan to disseminate, genetically characterize and evaluate the MAGIC lines in field trials through an evaluation network. This approach expands the trait data and will identify lines adapted to a range of production constraints (particularly stress tolerance) in Asia and Africa. The goal is to identify the best MAGIC lines with a combination of essential traits that are suitable for multiple environments.

**Table 2:** **Examples of** ***indica*** **MAGIC S4 lines exhibiting combination of traits**

S4 Designation	Bacterial blight strains				Blast^2^	Submergence tolerance (SES^3^)	Salt tolerance (SES^3^)	Grain length (mm)	Grain width (mm)	Amylose content (% by wt)	Gelatinisation temperature (DSC^4^)
	PXO61^1^	PXO86^1^	PXO99^1^	PXO341^1^							
**IR93326:8-B - 3–22 - 6**	MR	MS	S	MR	R	1	7	6.63	2.25	25.6	76.13
**IR93326:14-B - 7–22 - 7**	R	MS	MS	R	R	5	3	6.55	2.24	25.3	76.4
**IR93326:18-B - 12–7 - 15**	R	MS	S	R	S	1	7	6.58	2.14	25.7	76.6
**IR93328:10-B - 8–12 - 19**	MR	MS	MS	MR	R	1	7	6.24	2.07	23.8	76.61
**IR93328:11-B - 12–17 - 25**	MR	MS	MS	MR	R	5	7	6.98	1.88	23.8	76.83
**IR93328:23-B - 23–11 - 17**	R	MS	MS	MR	R	1	7	6.4	1.83	23	77.28
**IR93328:32-B - 21–8 - 5**	MR	MS	MS	MR	R	5	5	5.57	1.93	23.5	76.64
**IR93328:37-B - 6–13 - 19**	MR	MS	MS	MR	R	1	5	6.22	1.91	21.7	76.94
**IR 93329:1-B - 5–13 - 15**	R	MS	MS	R	R	1	7	5.55	2.34	22.2	76.82
**IR 93329:6-B - 19–18** - **13**	R	R	MR	R	R	1	5	5.59	2.37	26.4	69.03
**IR 93329**:**8**-**B** - **14**–**17** - **16**	MR	MS	S	R	R	5	7	6.2	2.18	21.8	77.39
**IR 93329**:**11**-**B** - **18**–**22** - **16**	R	R	MR	R	R	1	7	5.71	2.33	23.1	67.43
**IR 93329**:**18**-**B** - **14**–**16** - **17**	R	MS	MS	R	R	5	7	5.72	2.32	25.2	76.31
**IR 93329**:**25**-**B** - **26**–**16** - **12**	R	MS	MS	R	R	1	7	5.41	2.32	26.7	68.73
**IR 93329**:**26**-**B** - **17**–**19** - **12**	MR	MS	MS	MR	R	5	7	6.65	2.36	24.1	76.14
**IR 93329**:**29**-**B** - **1**–**23** - **7**	R	S	S	MR	R	1	7	6.63	2.19	24.2	67.85
**IR 93329**:**36**-**B** - **21**–**9** - **14**	R	MS	MS	R	R	5	3	6.85	2.25	22.4	76.7
**IR 93329**:**37**-**B** - **25**–**13** - **19**	R	MS	MS	R	S	5	7	6.29	2.08	25.1	75.83
**IR 93329**:**42**-**B** - **14**–**16** - **18**	R	R	MR	R	R	5	7	6.15	2.2	26.3	68.06
**IR 93330**:**4**-**B** - **20**–**19** - **13**	R	MS	S	MR	R	1	7	6.39	2.12	23.5	79.69

^1^ Reaction to bacterial blight strains, *R*, Resistant; *MR*, Moderately resistance; *MS*, Moderately susceptible; *S*, Susceptible.

^2^Under field condition.

^3^*SES*, Standard Evaluation System.

^4^*DSC*, Differential Scanning Calorimetry.

Testing the diverse AILs across a wide range of environments will provide a better understanding of the extent of adaptation for these highly recombined MAGIC populations as well as of G × E interactions. Fine mapping of the AILs from the *indica* MAGIC population will be used to develop trait-specific SNP markers for application in breeding programs. All data (genotype and phenotype) – including those collected from multi-environment trials would be stored in a database to enable breeders to improve their selections. Thus the different MAGIC populations will provide the foundation for accelerating progress in breeding programs through identification of beneficial alleles at key genetic loci, as well as superior combinations of stress tolerance in the best MAGIC lines for the breeding programs.

## Methods

### Phenotyping the subset of 200 lines and 8 parents

The purpose of the phenotyping experiments was to scan the population for multiple traits and combinations of traits captured at a relatively early generation (S4). Due to the limited amount of seeds, phenotyping experiments were not replicated. Based on the preliminary results from this study detailed phenotyping will be undertaken at the S8 stage when more seeds are available for replicated tests.

#### Blast disease

Phenotyping for resistance to blast disease was conducted in the Blast Nursery at IRRI. The blast nursery is a hot spot for blast disease in the Institute and is routinely used to screen lines for resistance to the natural isolates at the nursery. IR72 and Co39 lines known to be susceptible to blast disease were used as controls. A mixture of five susceptible varieties, called spreader rows, were planted around the plots to maintain the pathogen diversity and to enhance natural infection. Seeds were sown at a high density (about 5 gm per line). Humidity was maintained in the seedling beds by a sprinkler system and covering the plots with a plastic sheet in the evenings and removing them in the morning. Previously infected plants from spreader rows were used to infect the new batch of spreader rows. Disease then spread from the border rows to the test lines. The percent diseased leaf area (DLA) was recorded when the susceptible check was dead usually 14 days from date of infecting spreader rows. Overall DLA per line was assessed using the method described by Notteghem et al. (1981). As a large number of lines exhibited resistance the experiment was repeated to make certain that they were not escapes.

#### *Bacterial blight*(strains PXO61, PXO86, PXO99 and PXO341)

Seedlings were raised in the greenhouse up to 18 days and transplanted in the screen house. They were screened for resistance to 4 strains of *Xanthomonas oryzae* (PXO61, PXO86, PXO99 and PXO341) (Cottyn and Mew 2004). Two plants were screened per race and in each plant 5 leaves were inoculated. Plants were inoculated by the leaf clipping methods (dipping the scissors in a tube of inoculum and clipping the leaf tip) (Kauffman et al. 1973) 45 days after sowing (maximum tillering stage). Two weeks post inoculation the diseased leaf area was measured for each of the inoculated leaves and mean value was used for analysis.

#### Salinity

Salinity tolerance screening of the S4 *indica* MAGIC subset was done in a controlled environment condition at the IRRI phytotron facility. The test was conducted using salinized nutrient solution. FL478 (IR66946-3B-178-1-1) was the tolerant check and the sensitive check was IR29. The seeds were pre-germinated by soaking in water and incubated for 24–48 h at 35°C. Pre-germinated seeds were placed on a styrofoam seedling float that was placed on a tray filled with water. The styrofoam float had wells with a mesh at the bottom. In each well two seeds were placed and 4 wells were used per line. After 3 days, when seedlings were well established, the water was replaced with salinized Peter’s (20-20-20) water soluble fertilizer as hydroponic solution at a rate of 1 g per liter water. To this 200 mg ferrous sulphate per liter of water was added. The solution was salinized to EC = 12 dSm-^1^ by adding NaCl and adjust pH to 5.1 which was maintained over the period. An overall score was given per line. After two weeks in EC 12dSm^-1^ the visual reactions of plant to salinity stress were evaluated using the Standard Evaluation Score for salinity tolerance (1 = very tolerant, and 9 = very susceptible) (IRRI 1996).

#### Submergence

Seeds were directly sown at a high density (5 gm per line) in the submergence field ponds at IRRI. These ponds are specially designed to hold water up to 1.5 m in depth. Two week old seedlings were subjected to complete submergence for 16 days after which the water was drained, based on the inspection of the susceptible check IR42. Survival of all lines was scored 14 days after drainage using a five-class visual score (IRRI 1996).

#### Grain quality

Grain quality of the S4 grain (about 20 gm per line) of the subset was evaluated at the Grain Quality, Nutrition and Postharvest Center at IRRI using standard methods to test for physical traits (e.g. grain length (mm) and width (mm)) and other properties like amylose content (% by weight) based on the American Association of Cereal Chemists Method 61–03 (AACC, 1999) and gelatinization temperature by DSC (Differential Scanning Calorimetry) (Cuevas et al. 2010).

### Genotyping

#### Applying genotyping by sequencing (GBS) for genome-wide SNP scans in S4 indica MAGIC subset

The genotyping by sequencing approach used previously to genotype bi-parental populations (Elshire et al. 2011); (Huang et al. 2010) was used in this study. The DNA from a subset set of 200 S4 lines and 8 founders were digested using *Ape* KI and barcoded following the protocol of (Elshire et al. 2011). Sets of 96 samples per lane were sequenced on an Illumina HiSeq at Cornell University. SNP calls were made using Nipponbare as reference.

### GWAS of S4 *indica* MAGIC (SSD)

A subset of 200 *indica* MAGIC S4 lines along with 8 founders was used for association mapping of selected traits referred to as *indica* MAGIC subset. Results from this mapping exercise must be considered preliminary as only a small population was used, limiting the power to detect small QTLs reliably. Even so, the association mapping results detected highly significant major genes and QTLs and confirmed the presence of several known loci.

The SNP marker data was obtained from GBS of a subset of the *indica* MAGIC population (200 S4 lines and 8 founders). After processing the raw data through the TASSEL GBS pipeline, there were 109,610 SNP markers. There were a large proportion of missing calls along with many heterozygous SNPs detected in the population. The Trait Analysis by Association Evolution and Linkage (TASSEL) program (Bradbury et al. 2007) was used for the association analysis. As the confidence level of calling heterozygous state (hets) was low all were considered as missing data. Only 0.65% of the data points were hets. The sites were filtered at a maximum count of 130 of 217 which accounts for sites where 60% of the lines have a call and a minimum frequency of 0.05 for the minor allele. The above criteria resulted in 17,387 filtered sites which were used for genome wide association mapping. The 17,387 sites included missing calls. The missing calls were not imputed for GWAS. In this study we have used only the progenies for analysis. Thereby we have in total 200 S4 MAGIC lines represented in the analysis. MAGIC populations are considered to have little to no population structure due to the high levels of recombination (Mackay and Powell 2007), although others are of the opinion that the minimal structure should be accounted for. In this study the mixed linear model (MLM) was used to associate the markers to the traits.

#### Mixed linear model (MLM)

The mixed linear model uses both fixed and random effects which incorporates kinship among the individuals. Population structure in AILs and genome shuffling may need to be accounted for QTL mapping and its importance has been emphasized (Balding 2006; Peirce et al. 2008). For running the mixed linear model, a kinship matrix was generated using all SNP markers sites containing no heterozygous calls. The model for kinship matrix was set to model heterozygotes as related to homozygotes, which is the default setting although there were no heterozygotes in the dataset used for kinship. A united data file with the genotype and phenotype of the lines was created. The united file along with kinship matrix was used to analyze associations using MLM across 17,387 sites. The compression level was set to optimum level (Zhang et al. 2010) to reduce computation time. The MLM analysis gives 3 outputs the model statistics, model effects and the compression if applied. The model statistics gives the p-value from the F-distribution for which we used a cut-off p < 0.0001 and this output also gives the R^2^ for the marker. The model effects indicate the allelic states and effect of the state.

#### General linear model (GLM)

Analysis for the GLM was conducted by combining the genotype data for 17,387 SNP markers and the phenotype data and using the united dataset for analysis. One thousand permutation tests per marker were run to determine a significant threshold. The GLM analysis uses a least squares fixed effects linear model and gives two outputs (a) GLM marker test and (b) the allele estimates.

The GLM marker test gives the marker-p value and the marker R^2^ values. In this study we set a significant threshold of p < 0.0001. The q-values were used to eliminate false positives. Q-values were calculated using the q-value package in R (Storey 2003).

#### qvalue

The false positives were eliminated by calculating the q-value using the R-package “q value” (Storey 2003). The raw p-values derived from GLM analysis was loaded in R and the lambda range/tuning parameter was set between 0–0.9 by 0.05 and the smoother pi_0 method. The robust method was used and False Discovery Rate (FDR) level was set at 0.05. In the current study, a q-value of < 0.05 was generally used as cut-off. In our data set we had 17,387 markers, the p-values of each of these markers was used to derive a q-value for each of the markers. We set q-value cut off at 0.05 which means 5% of the markers with a q-value of <0.05 are false positives.

## Electronic supplementary material

Additional file 1: Figure S7: Manhattan plots of GLM analysis. Blast disease - Manhattan plot (GLM) showing GWA and highlighting significant associations near previously detected QTLs on chromosomes 2, 3, 9 and 10; x axis – position on chromosomes 1 to 12; y-axis (-) Log p-value of markers; solid line – p < 0.0001. **Figure S8**: Bacterial blight - Manhattan plots (GLM) showing GWA to bacterial blight strains (a) PXO61 and (b) PXO341(c) PXO86 and (d) PXO99. Note the high level of association with SNPs on chromosome 11 (figures a and b) pointing to *Xa4* and *xa5* on chromosome 5 (figure c). GWAS detects both *Xa4* and *xa5* (figure d) in response to PXO99. x axis – position on chromosomes 1 to 12; y-axis (-) Log p-value of markers; solid line – p < 0.0001. **Figure S9**: Abiotic stress - Manhattan plots (GLM) showing GWA to (a) salt tolerance and (b) submergence tolerance. Note the association on chromosome 1 - Saltol to salt tolerance and detection of the *SUB1* locus on chromosome 9 in response to submergence. x axis – position on chromosomes 1 to 12; y-axis (-) Log p-value of markers; solid line – p < 0.0001. **Figure S10**: Grain quality - Manhattan plots (GLM) showing GWA for (a) amylose content – *waxy* chromosome 6 (b) grain length GS3 on chromosome 3 (c) grain width GB* QTL for grain breadth on chromosome 7 (Redoña and Mackill1998), and (d) gelatinization temperature *SSIIa* chromosome 6. x axis – position on chromosomes 1 to 12; y-axis (-) Log p-value of markers; solid line – p < 0.0001. (DOCX 2 MB)

Additional file 2: A list of selected markers that were significantly associated with the trait (by using the mixed linear model) has been provided as an additional file. (XLSX 14 KB)

Below are the links to the authors’ original submitted files for images.

Authors’ original file for figure 1

Authors’ original file for figure 2

Authors’ original file for figure 3

Authors’ original file for figure 4

Authors’ original file for figure 5

Authors’ original file for figure 6
