# Supplementary figures and images for: Multi-parent advanced generation inter-cross (MAGIC) populations in rice: progress and potential for genetics research and breeding
Source: Rice (N Y). 2013 May 6;6:11. doi: 10.1186/1939-8433-6-11 (PMC4883706; doi:10.1186/1939-8433-6-11)

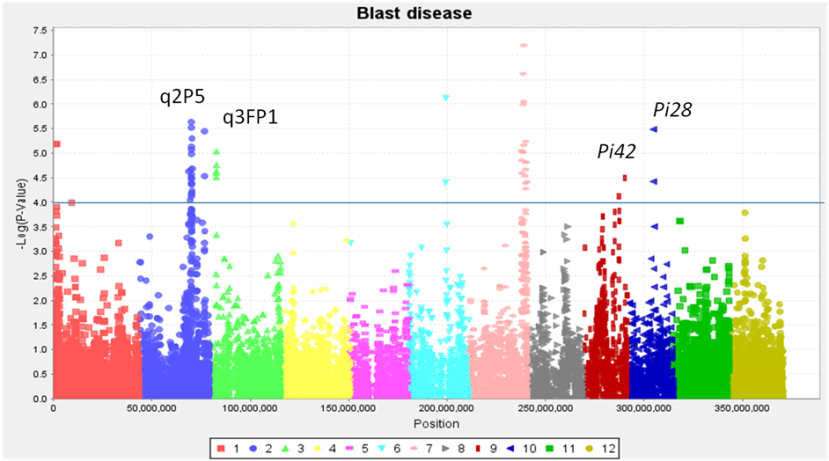


Figure 7 (supp)


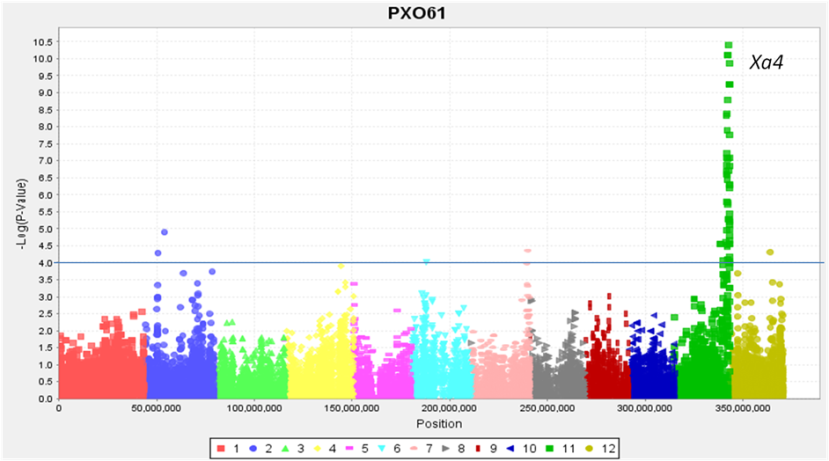

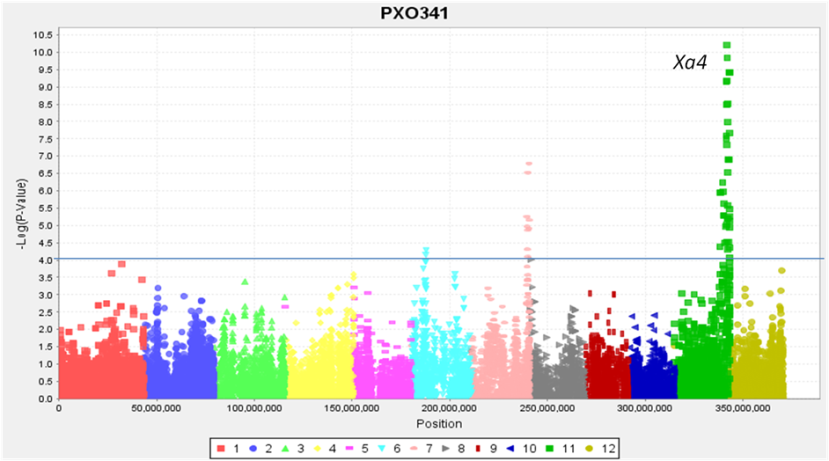

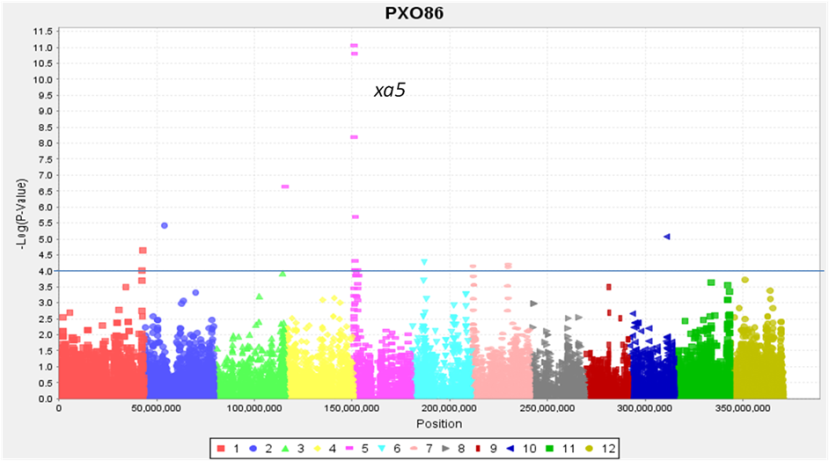

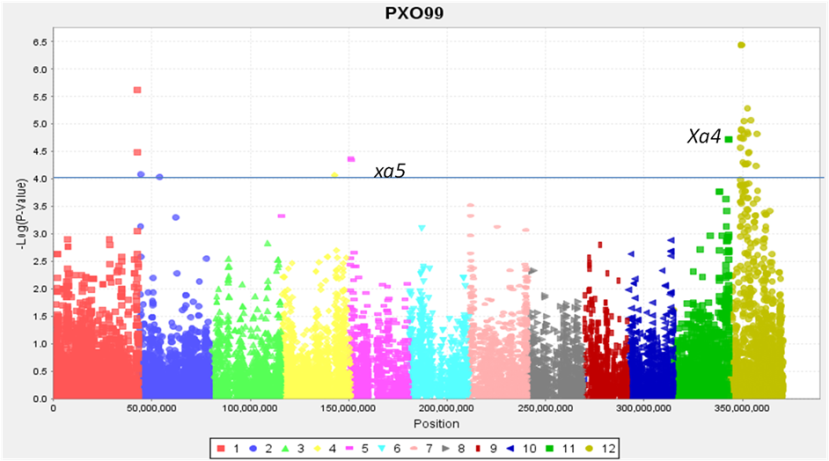


b

a

c

d

Figure 8 (supp.)


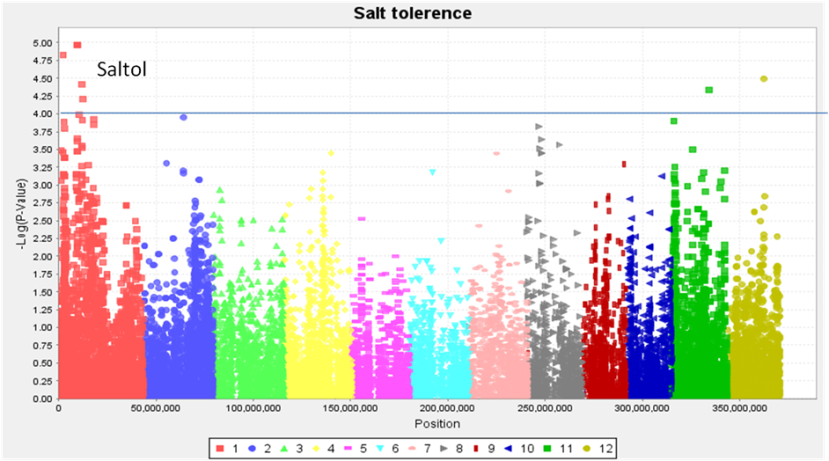


b

a


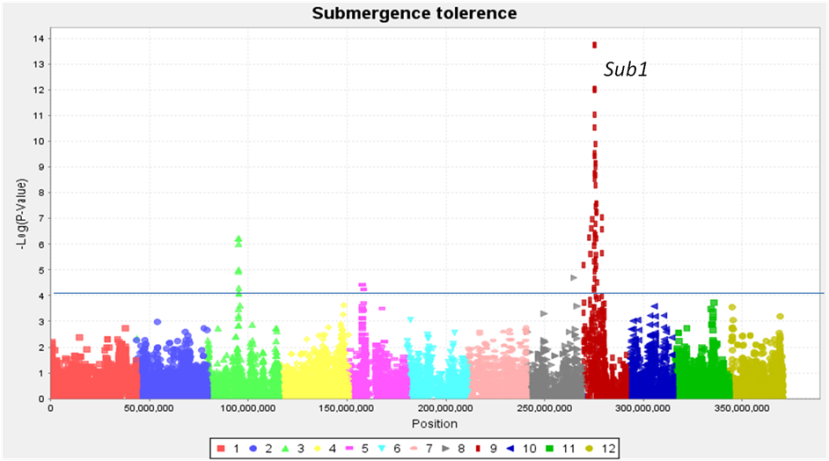


Figure 9 (supp.)


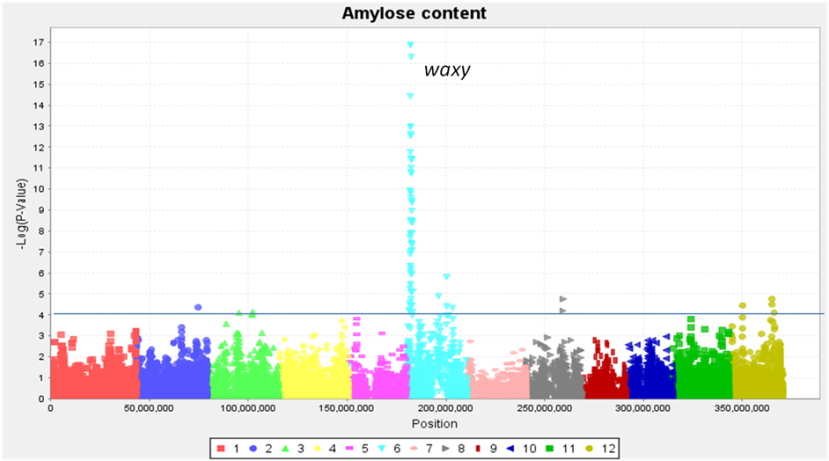

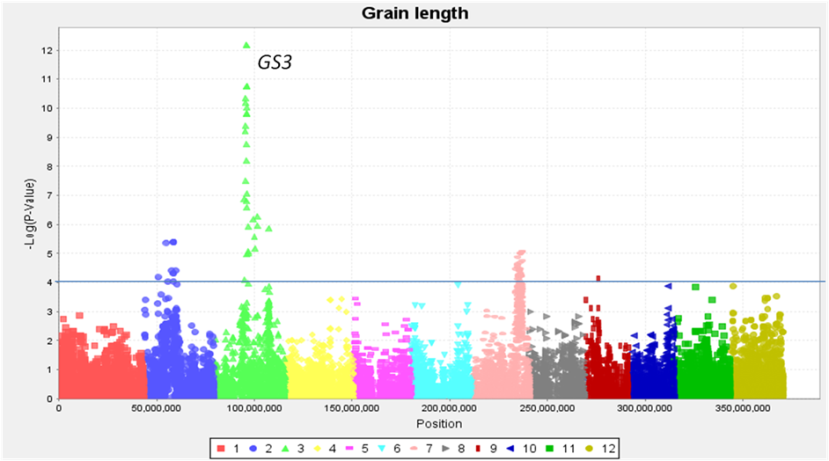


d

c

b

a


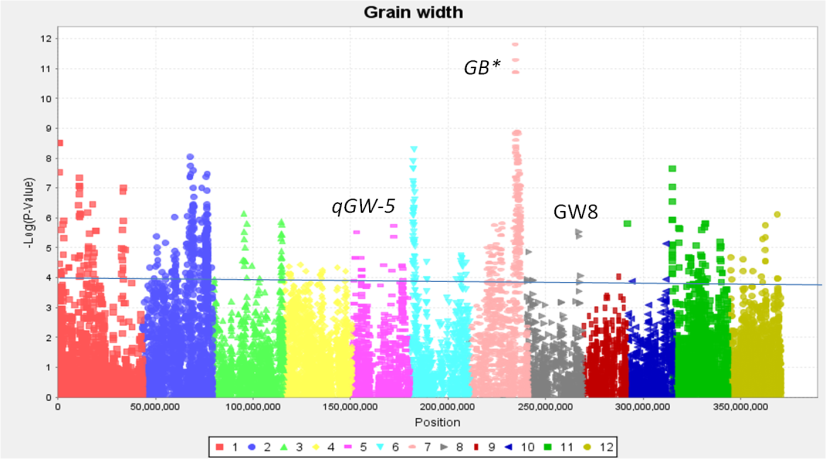


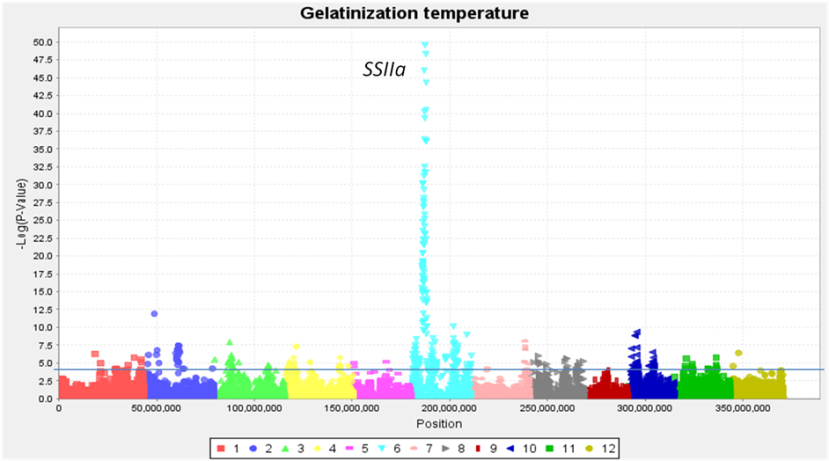


Figure 10 (supp.)

Supplement: Supplementary file 1 — Additional file 1: Figure S7: Manhattan plots of GLM analysis. Blast disease - Manhattan plot (GLM) showing GWA and highlighting significant associations near previously detected QTLs on chromosomes 2, 3, 9 and 10; x axis – position on chromosomes 1 to 12; y-axis (-) Log p-value of markers; solid line – p < 0.0001. Figure S8: Bacterial blight - Manhattan plots (GLM) showing GWA to bacterial blight strains (a) PXO61 and (b) PXO341(c) PXO86 and (d) PXO99. Note the high level of association with SNPs on chromosome 11 (figures a and b) pointing to Xa4 and xa5 on chromosome 5 (figure c). GWAS detects both Xa4 and xa5 (figure d) in response to PXO99. x axis – position on chromosomes 1 to 12; y-axis (-) Log p-value of markers; solid line – p < 0.0001. Figure S9: Abiotic stress - Manhattan plots (GLM) showing GWA to (a) salt tolerance and (b) submergence tolerance. Note the association on chromosome 1 - Saltol to salt tolerance and detection of the SUB1 locus on chromosome 9 in response to submergence. x axis – position on chromosomes 1 to 12; y-axis (-) Log p-value of markers; solid line – p < 0.0001. Figure S10: Grain quality - Manhattan plots (GLM) showing GWA for (a) amylose content – waxy chromosome 6 (b) grain length GS3 on chromosome 3 (c) grain width GB* QTL for grain breadth on chromosome 7 (Redoña and Mackill1998), and (d) gelatinization temperature SSIIa chromosome 6. x axis – position on chromosomes 1 to 12; y-axis (-) Log p-value of markers; solid line – p < 0.0001. (DOCX 2 MB) [file 12284_2013_50_MOESM1_ESM.docx]

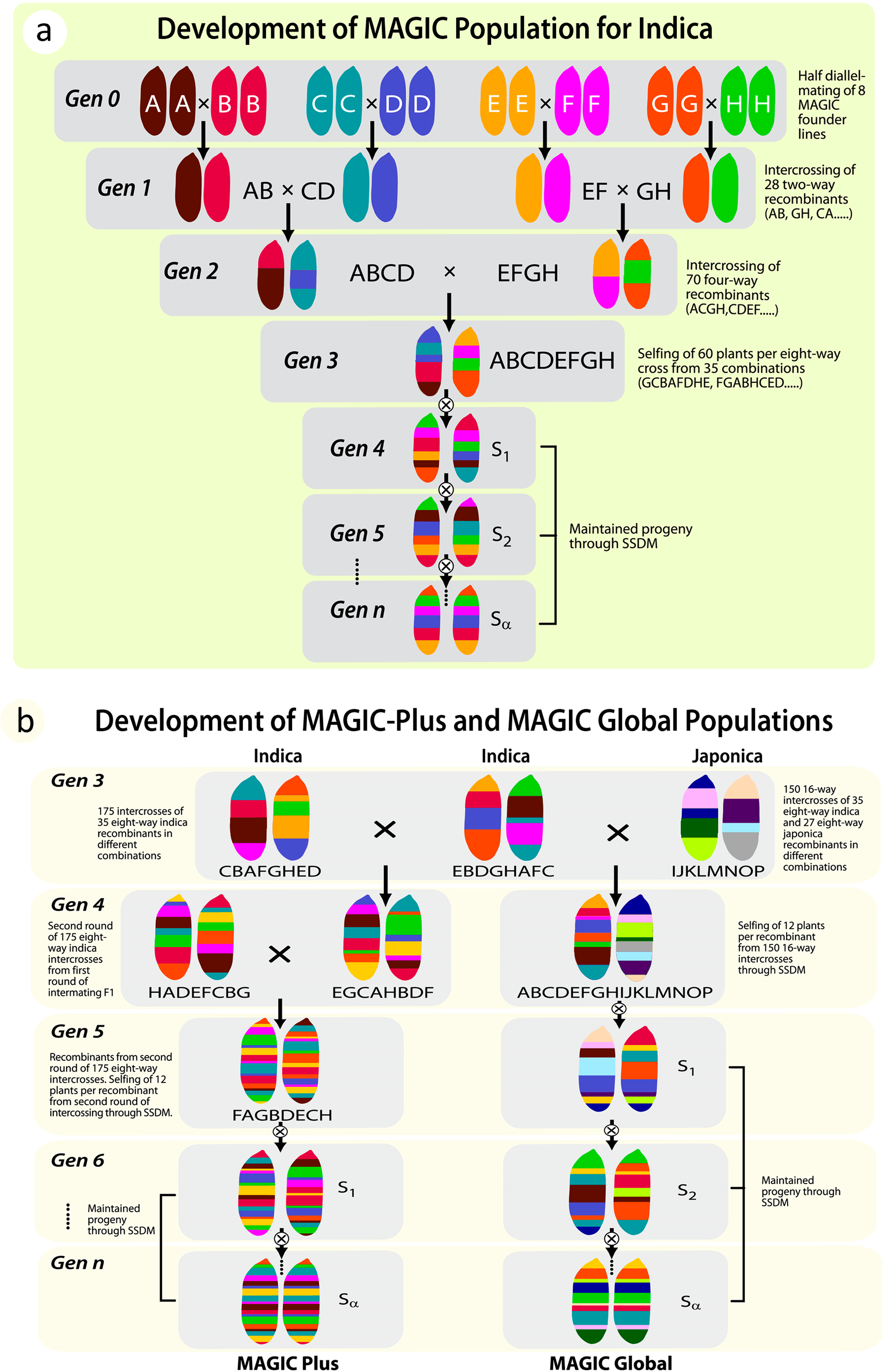

Supplement: Supplementary file 3 — Authors’ original file for figure 1 [file 12284_2013_50_MOESM3_ESM.tiff]

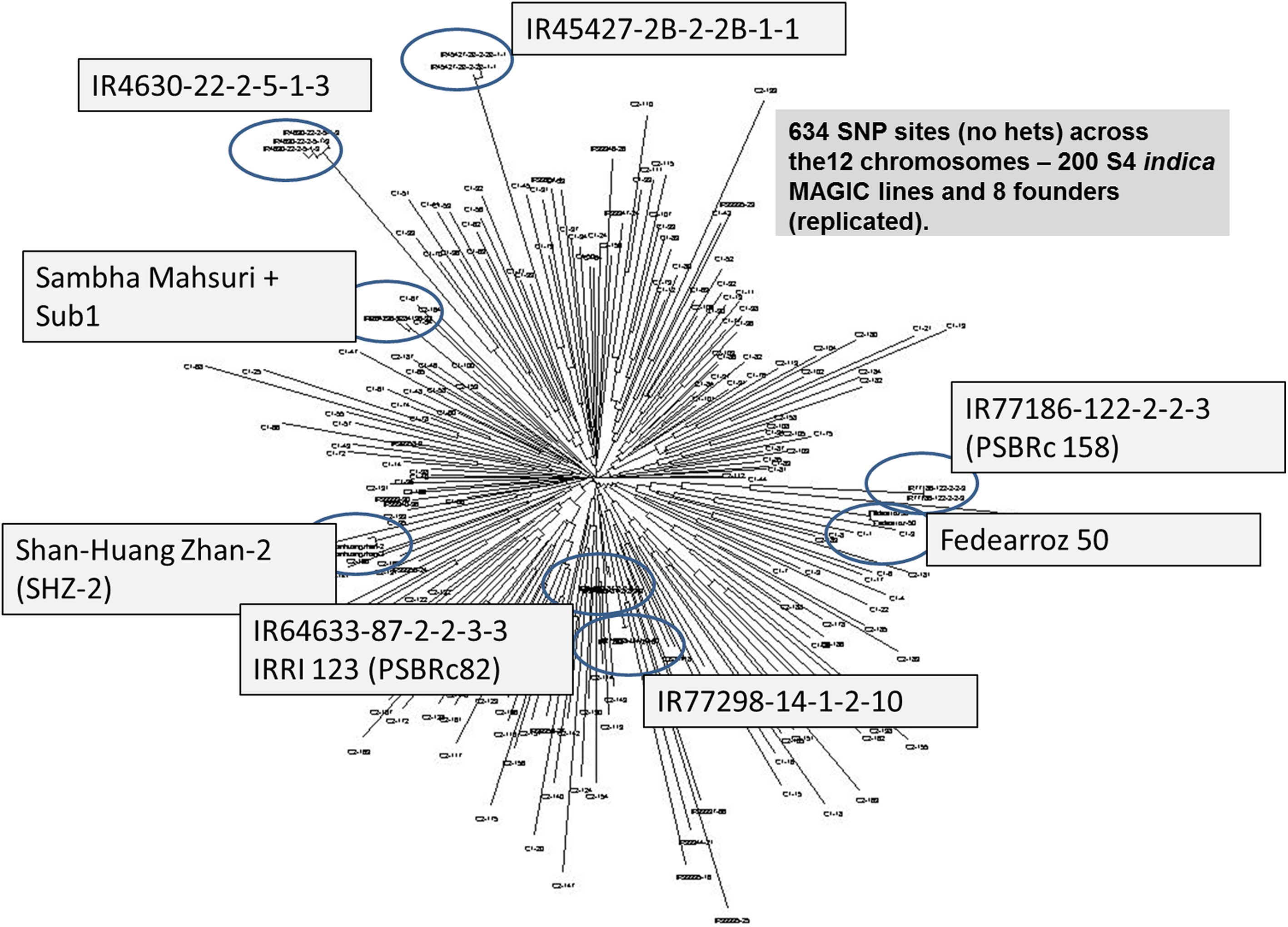

Supplement: Supplementary file 4 — Authors’ original file for figure 2 [file 12284_2013_50_MOESM4_ESM.tiff]

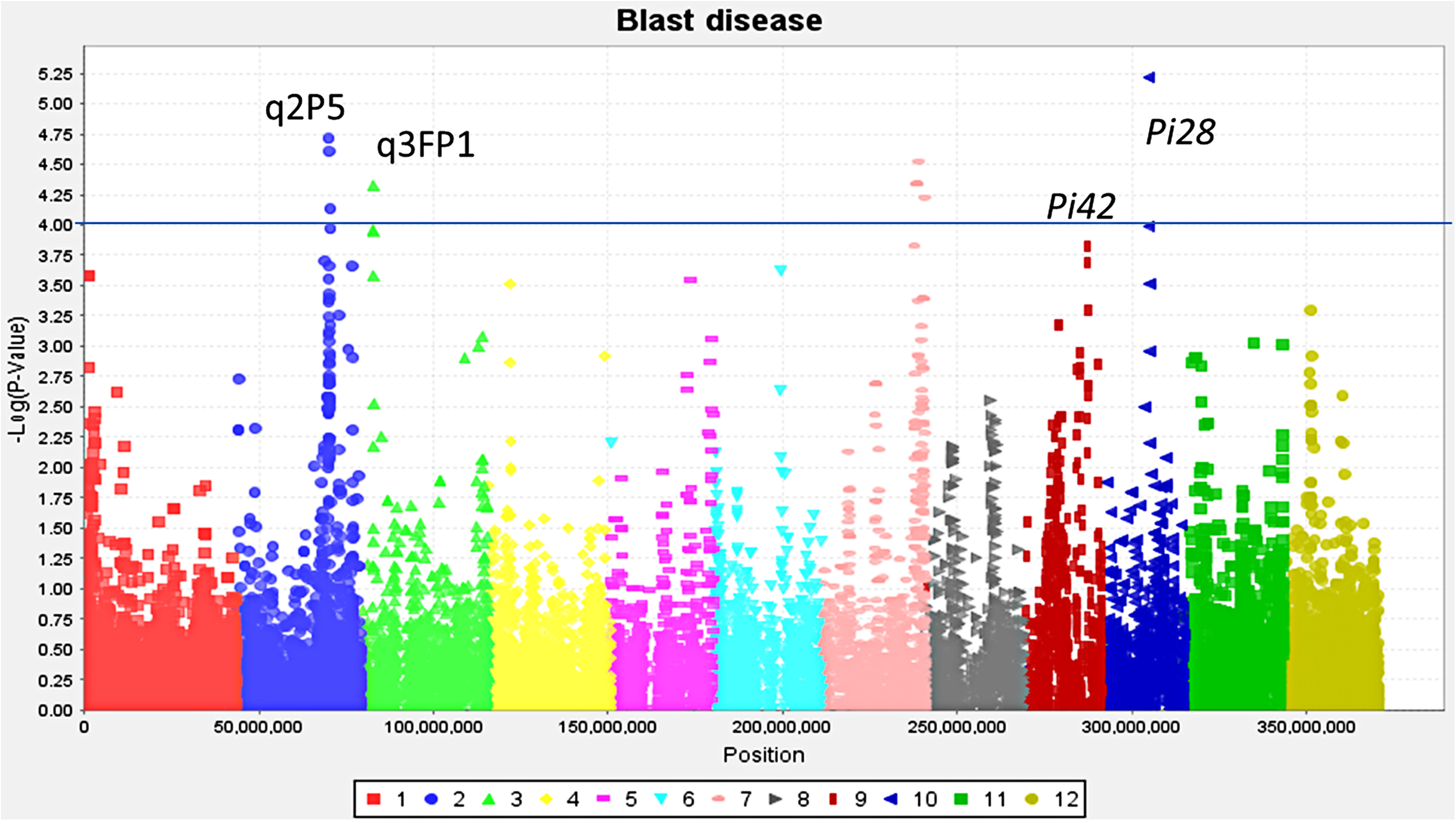

Supplement: Supplementary file 5 — Authors’ original file for figure 3 [file 12284_2013_50_MOESM5_ESM.tiff]

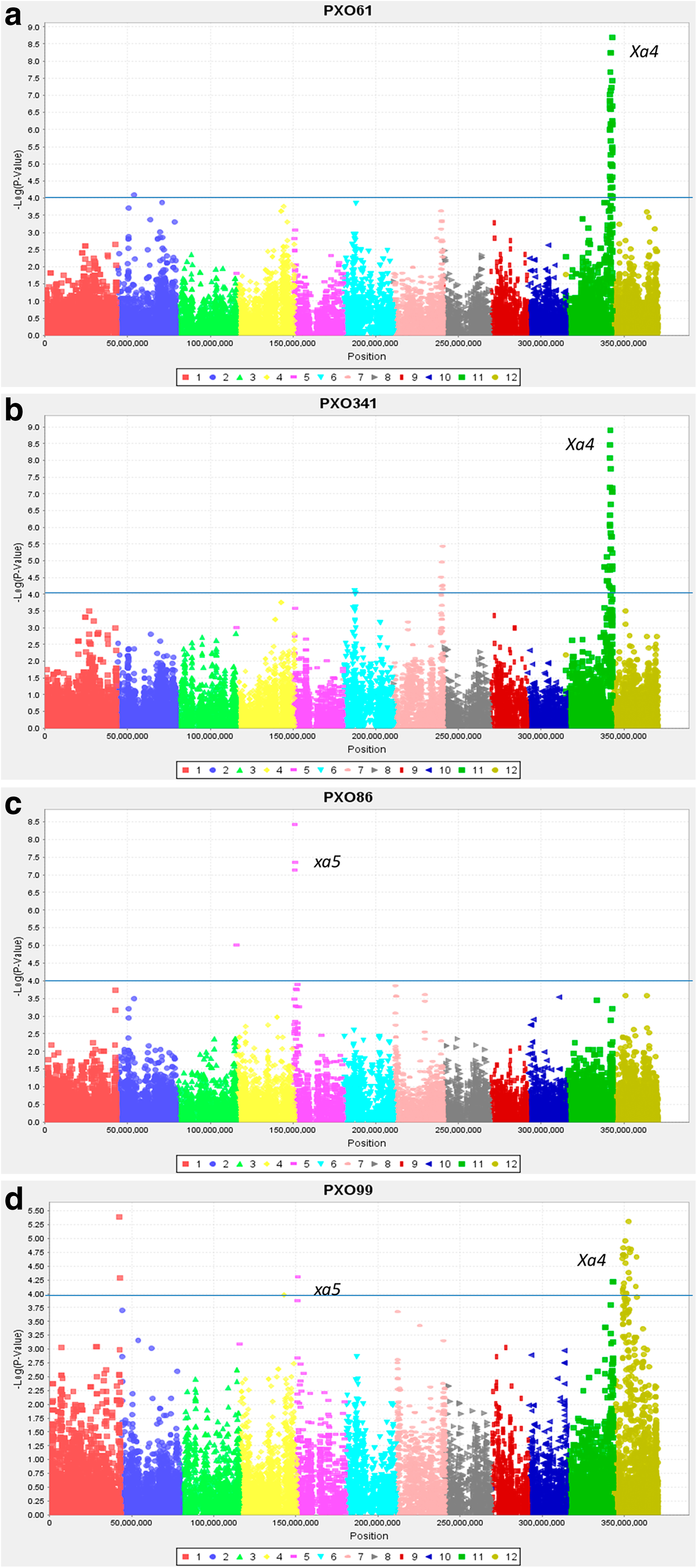

Supplement: Supplementary file 6 — Authors’ original file for figure 4 [file 12284_2013_50_MOESM6_ESM.tiff]

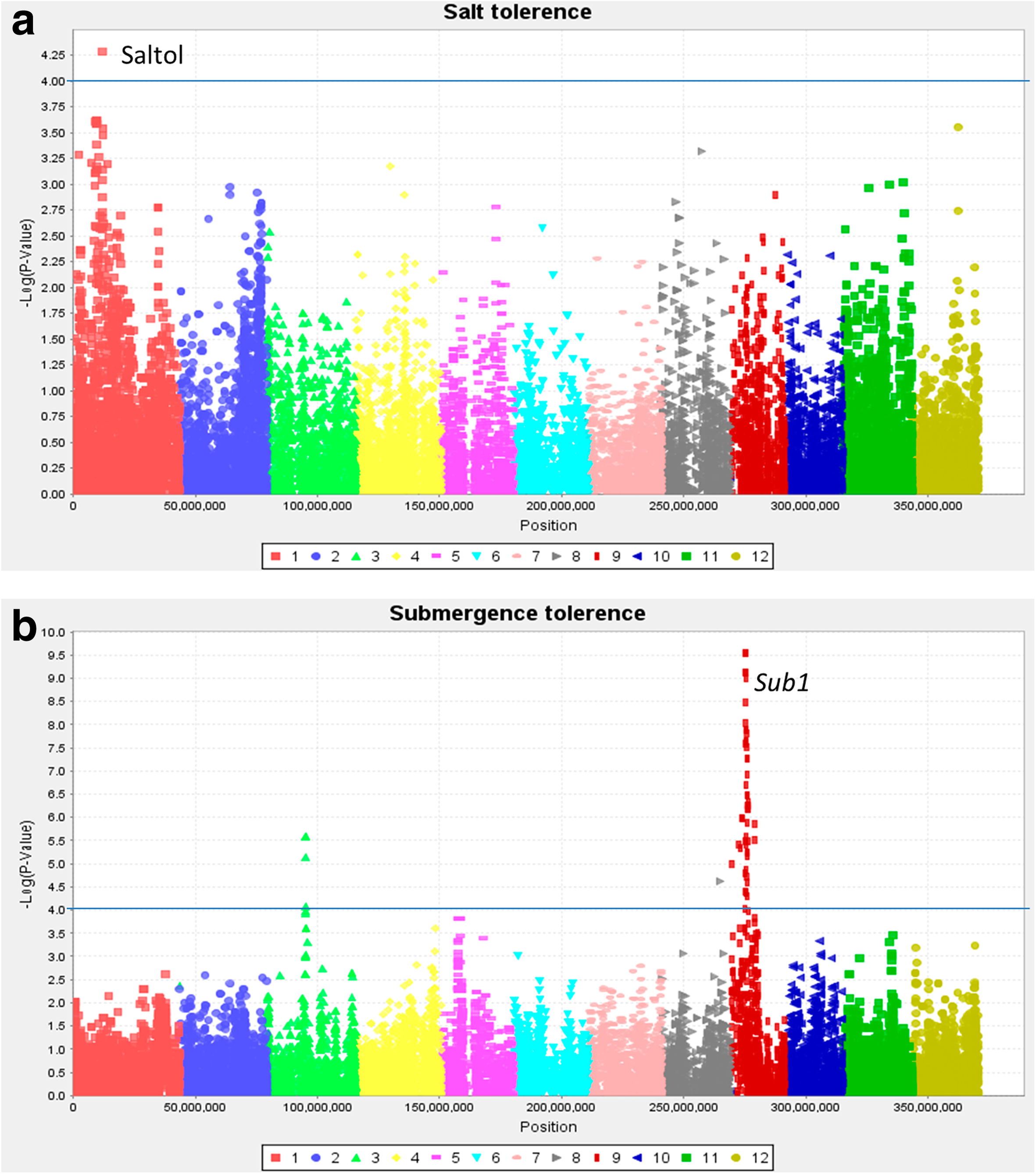

Supplement: Supplementary file 7 — Authors’ original file for figure 5 [file 12284_2013_50_MOESM7_ESM.tiff]

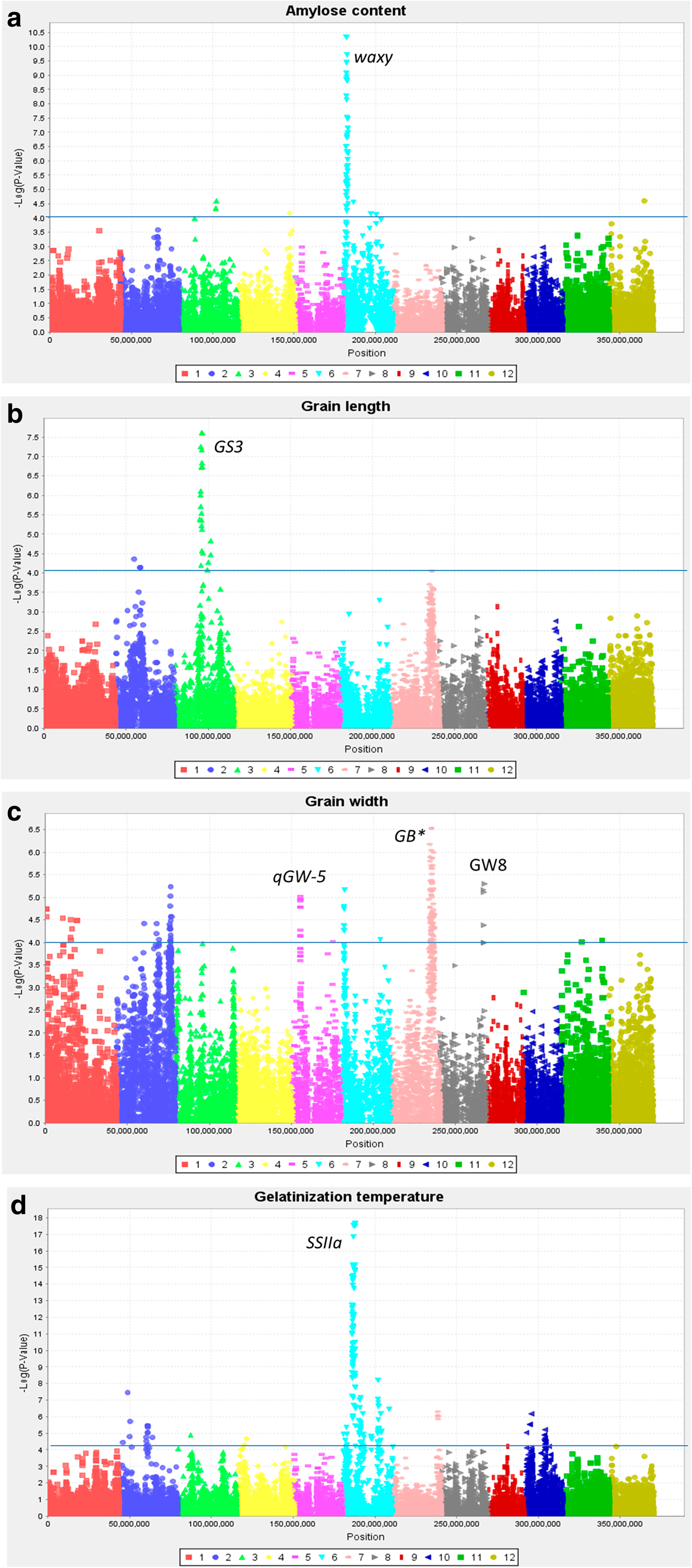

Supplement: Supplementary file 8 — Authors’ original file for figure 6 [file 12284_2013_50_MOESM8_ESM.tiff]
